# Neo-Antigen mRNA Vaccines

**DOI:** 10.3390/vaccines8040776

**Published:** 2020-12-18

**Authors:** Arthur Esprit, Wout de Mey, Rajendra Bahadur Shahi, Kris Thielemans, Lorenzo Franceschini, Karine Breckpot

**Affiliations:** Laboratory for Molecular and Cellular Therapy (LMCT), Department of Biomedical Sciences, Vrije Universiteit Brussel, B-1090 Brussels, Belgium; arthur.esprit@vub.be (A.E.); wout.de.mey@vub.be (W.d.M.); Rajendra.Bahadur.Shahi@vub.be (R.B.S.); kris.thielemans@vub.be (K.T.); lorenzo.franceschini@vub.be (L.F.)

**Keywords:** cancer, neo-antigen, mRNA, vaccine, dendritic cell, T cell

## Abstract

The interest in therapeutic cancer vaccines has caught enormous attention in recent years due to several breakthroughs in cancer research, among which the finding that successful checkpoint blockade treatments reinvigorate neo-antigen-specific T cells and that successful adoptive cell therapies are directed towards neo-antigens. Neo-antigens are cancer-specific antigens, which develop from somatic mutations in the cancer cell genome that can be highly immunogenic and are not subjected to central tolerance. As the majority of neo-antigens are unique to each patient’s cancer, a vaccine technology that is flexible and potent is required to develop personalized neo-antigen vaccines. In vitro transcribed mRNA is such a technology platform and has been evaluated for delivery of neo-antigens to professional antigen-presenting cells both ex vivo and in vivo. In addition, strategies that support the activity of T cells in the tumor microenvironment have been developed. These represent a unique opportunity to ensure durable T cell activity upon vaccination. Here, we comprehensively review recent progress in mRNA-based neo-antigen vaccines, summarizing critical milestones that made it possible to bring the promise of therapeutic cancer vaccines within reach.

## 1. Introduction

Cancer immunotherapy aspires the selective destruction of cancer cells by the patients’ immune system. Therefore, the focus lies on inducing robust T cell-mediated anti-cancer immunity. In particular, stimulation of CD4^+^ T helper 1 (T_H_1) cells and cytotoxic T lymphocytes (CTLs) is envisaged, although activation of invariant natural killer T (iNKT) cells has been studied as well [1,2,3]. CD4^+^ T_H_1 cells and iNKT cells play a supportive role, ensuring that CTLs are properly activated upon interaction with professional antigen-presenting cells (APCs) that present cancer antigens in the context of major histocompatibility complex (MHC, human leukocyte antigen [HLA] in humans) class I and II molecules. Both cell types can moreover play a critical part in cancer rejection [4,5,6,7].

At present, numerous cancer vaccines have been developed. However, achieving clinical efficacy that exceeds 10% objective clinical responses has been challenging, with only a number of studies reporting on overall clinical benefit exceeding 25% [8,9]. Most of these vaccines were based on shared cancer antigens, including cancer-testis and differentiation antigens [10]. However, these antigens are not cancer-specific, not highly immunogenic and are subjected to central tolerance mechanisms. Therefore, it is thought that the full potential of cancer vaccines has not yet been reached due to the choice of the targeted antigens. Advances in next-generation sequencing (NGS), bioinformatics and peptidomics have enabled the identification of non-synonymous mutations and other alterations of the cancer cell genome (intron retention, indels, frameshifts, etc.), emerging as neo-antigens and resulting in the development of personalized vaccines [11]. Neo-antigens hold the promise of tumor specificity, therefore, this ensures on-target toxicity, without being off-tumor, and likely elicits high affinity T cells [12,13,14,15,16]. Recent clinical trials in, among others, melanoma and glioblastoma patients support further development of neo-antigen vaccines, as these show stimulation of de novo as well as existing neo-antigen specific T cells [17,18,19,20,21,22].

**Figure 1:**
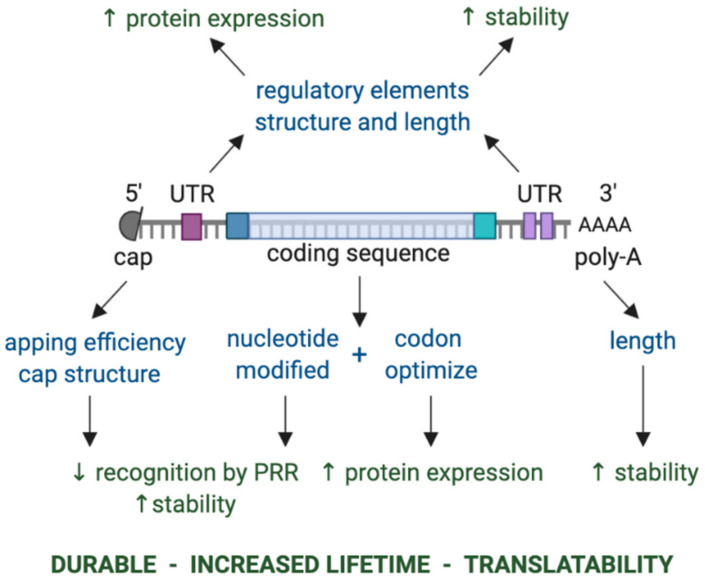
Key components of in vitro transcribed mRNA that determine the level and duration of expression of the encoded protein. The components that can be modulated are shown in blue, while the effect of modulating these components is shown in green. Abbreviations: 3′ poly-A, three prime polyadenylic acid tail; 5′ cap, five prime cap; PRR, pattern recognition receptor; UTR, untranslated region.

Because the majority of neo-antigens are unique to each patient’s cancer, a vaccine technology that is flexible and potent is required to develop personalized neo-antigen vaccines. In this regard, in vitro transcribed (IVT) mRNA has come to the forefront as a cost-effective modality to generate neo-antigen cancer vaccines that likely elicit strong anti-tumor immunity (Figure 1) [23,24]. Notably, IVT mRNA has different advantages over other vaccines, e.g., vaccines based on proteins/peptides, viral vectors or DNA. Firstly, IVT mRNA has a high safety profile. It is a non-infectious vehicle that does not integrate into the host genome; therefore, there is no risk of infection or insertional mutagenesis. Moreover, IVT mRNA is only transiently present in transfected cells, as it is quickly degraded by intracellular ribonucleases (RNases). Nevertheless, its lifetime can be modulated if needed through chemical modification and sequence engineering of the molecule [25,26,27,28,29,30,31]. IVT mRNA can elicit potentially harmful type I interferon (IFN) responses through interaction with pattern recognition receptors (PRRs). However, this is avoidable, as IVT mRNA can be rendered invisible for PRRs through the use of chemically modified nucleotides and incorporation of purification steps in the production protocol [32]. Protein expression efficiency from the mRNA template has vastly improved due to modifications to the mRNA molecule and sequence optimization, making it more stable, durable and easy to be translated into the encoded protein [25,26,32]. Moreover, through tweaking of the delivery method, delivery of the IVT mRNA in the cytoplasm, where it should be translated, is ensured [1,33,34,35,36]. Moreover, an immune response will only be generated against the mRNA encoded protein and not to the mRNA vector, as this mRNA is a minimal genetic vector. As a consequence, prime-boost vaccination regimes can be performed without loss of efficacy due to anti-vector immunity [37]. The manufacturing process of IVT mRNA is rapid, inexpensive and scalable, which from a biotech manufacturing perspective is appealing. In principle, facilities dedicated to mRNA production can quickly manufacture vaccines against any given target with a minimal adaptation of the processes and formulation. The latter is a major strength of mRNA for neo-antigen vaccine development.

Here, we review the state-of-the-art on the neo-antigen discovery and recent developments in mRNA-based neo-antigen vaccination. We describe the advantages of mRNA, the mechanisms of mRNA-based vaccines with their clinical development and propose future perspectives.

## 2. Neo-Antigens and Their Recognition by CD4^+^ or CD8^+^ T Cells

Neo-antigens can result from any change to the cancer genome that leads to the production of proteins with an altered protein sequence [14]. These DNA changes can include non-synonymous mutations or single nucleotide variants (SNV) [38,39,40], mutational frameshifts (insertions/deletions [INDELs]) [38,41,42], gene fusions [38,41], post-translational modifications that alter the amino acid sequence [38,42] and intron retention [42,43]. These neo-antigens are abnormal proteins that are subjected to cytosolic degradation regulated by ubiquitin and mediated by the proteasome [44]. As a result, short peptides (2–20 amino acid residues) are generated, of which some are transported to the endoplasmic reticulum for loading onto MHC-I molecules [45,46]. These peptide-loaded MHC-I molecules find their way through the Golgi apparatus to the cell membrane, resulting in antigen presentation and as a result the potential of recognition by the T cell receptor (TCR) of CD8^+^ T cells, which can then be activated to CTLs [47]. However, spontaneous activation of CTLs to neo-antigen derived epitopes (neo-epitopes) is not a frequent event [48].

Neo-epitopes that are restricted to MHC-II, antigen-presenting molecules that are mainly expressed on professional APCs, including dendritic cells (DCs), macrophages and B cells [49], have been identified in various mouse tumor models [50] as well as in cancer patients [51]. These MHC-II restricted neo-epitopes are presented to the TCR of CD4^+^ T cells, which upon activation can adopt various functions. Although a substantive CD4^+^ T cell response to a given neo-epitope is rather rare (~0.5% of neo-epitopes) [51], it is contended that neo-epitope-specific CD4^+^ T cells aid cancer regression in various ways, among others through direct killing of cancer cells and by supporting the priming, function and tumor infiltration of cancer-specific CD8^+^ T cells [52]. The observation that infusion of ex vivo expanded CD4^+^ T cells that recognized a neo-epitope derived from the mutated *ERBB2IP*, a gene encoding the ERBB2 interacting protein, into a patient with metastatic cholangio-sarcoma-induced significant tumor regression supports the importance of CD4^+^ T cells during the anti-cancer immune response [53]. This notion is not new and has been considered for a long time during the development of cancer vaccines in which non-mutated tumor-associated antigens (TAAs) were used [2,54,55].

Leng et al. [56] reviewed the immunogenicity of neo-antigens, linking the characteristics of immunogenic neo-epitopes to host exposure to microbes, and putting forward the hypothesis that pre-existing heterologous memory T cell immunity in cancer patients is determining the immunogenicity of neo-epitopes. It was shown earlier for TAAs that cross-reactivity, where a TAA shares sequence or structural similarity with a microbial antigen and as a result is recognized by the TCR of the microbe specific T cells, contributes to tumor control [57,58,59]. It was shown in melanoma patients treated with anti-CTLA-4 antibodies that patients with long-term clinical benefit shared neo-antigens with conserved tetrapeptide sequences homologous to microbial antigens, although studies confirming this finding have not yet been published [60]. Nonetheless, case reports support the idea that neo-antigens with considerable homology to microbial antigens are immunogenic [15,61]. Stronger evidence comes from an algorithm that scores the immunogenicity of neo-antigens based on their sequence homology with microbial antigens and the predicted affinity of the resulting neo-epitope for MHC binding when compared to the wild type epitope. This algorithm was able to divide pancreatic cancer patients into short and long-term survivors; therefore, it was able to use neo-antigens as predictive markers [61]. However, in other studies, such homology between neo-antigens and microbial antigens was not observed, suggesting that sequence homology to microbial antigens is not an absolute requirement for the neo-antigen’s immunogenicity [62,63]. The recent TESLA (Tumor Neo-antigen Selection Alliance) report describes key parameters that determine the immunogenicity of neo-epitopes. According to this study, immunogenic epitopes are characterized by low hydrophobicity, a strong affinity for binding to MHC proteins with a long half-life, high expression and high foreignness [64]. Therefore, it is safe to state that neo-epitopes should be similar to their wild type epitope with respect to amino acids that anchor the peptide to the binding groove of the MHC protein, while being divergent from their wild type epitope in terms of amino acids that are exposed for contact with the TCR, as this would allow the T cell to recognize the neo-epitope as non-self, thereby fulfilling the first requirement for T cell activation.

## 3. Identification and Validation of Neo-Antigens

**Figure 2:**
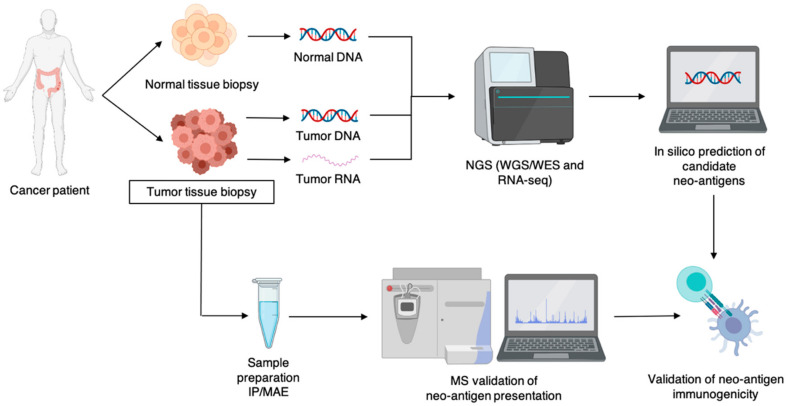
Schematic representation of the workflow for neo-antigen identification. Abbreviations: IP, immunoprecipitation; MAE, mild acid elution; MS, mass spectrometry; NGS, next-generation sequencing; WGS, whole exome sequencing; WES: whole exome sequencing.

The first reports on identification of neo-epitopes after studying T cell responses in both mouse [65,66,67] and human [68,69,70] cancer are over two decades old. These reports delivered a proof-of-concept that neo-antigens can serve as a target for tumor cell recognition; however, they provided little insight into straightforward strategies for neo-antigen/epitope identification. The recent advent of the high-throughput genome and peptidome analysis with sophisticated computational algorithms has paved the way for rapid neo-epitope identification and accurately predicting its affinity to HLA molecules as well as its immunogenicity (Figure 2) [11]. Despite these advances, several hurdles are yet to be overcome to implement neo-antigens into routine research and daily clinical practice. These hurdles are related to technical aspects, such as source, quantity and quality of tumor material for sequencing, and algorithms used for neo-antigen discovery and its affinity prediction [71,72,73] as well as to biological aspects, such as polymorphism of potential antigens and HLA molecules, gaps in our knowledge on HLA binding motifs for less frequent HLA alleles and heterogeneity of tumors [74]. Therefore, unmet needs are the availability of large training datasets for less frequent HLA alleles and high throughput assays to study the presentation and immunogenicity of predicted neo-epitopes.

In the past decade, NGS has facilitated the use of whole genome/exome sequencing and RNA sequencing, enabling rapid identification of non-synonymous mutations resulting in neo-antigens, while also allowing HLA allele genotyping. The use of NGS has contributed to the clarification of the origin of neo-antigens, showing that neo-antigens can arise from SNVs, INDELs, gene fusions and even non-coding regions of the genome [75]. For effective neo-antigen identification, NGS is combined with in silico T cell epitope prediction algorithms that assess the HLA binding affinity of the potential neo-antigen [38]. Two strategies have been explored in neo-antigen prediction; one method uses a stepwise analysis-based filtering strategy, while the other uses an integrative scoring-based strategy. The former implements a number of filtering steps based on the cut-off of certain metrics, such as binding affinity, sequence coverage and gene expression. The latter assigns a score based on certain characteristics of the neo-antigen, later used to empirically determine the immunogenicity [76]. As computational algorithms continue to improve, multiple of them have been made available on/off-line, e.g., IEDBtools, MHCflurry and MHCnuggets [38]. However, the use of NGS combined with bioinformatics prediction algorithms does not confirm whether candidate neo-antigens are presented in the corresponding HLA molecules. With this, it is important to mention that these predictions—as a stand-alone method for neo-antigen identification—are rather insufficient. Therefore, validation of the presentation and T cell reactivity of NGS-identified ‘candidate’ neo-epitopes is required [77,78]. The need for validation of neo-epitope presentation and immunogenicity is further evident from a number of studies that show lack of recognition by T cells for a significant portion of in silico selected candidate neo-antigens [79,80,81].

Mass spectrometry (MS) is an interesting strategy to examine and validate the presentation of neo-epitopes in HLA molecules. MS is an advanced analytical method for the detection of ionized peptides, intact proteins and small molecules. Efforts have been made to further improve MS by, for example, prefixing it to high pressure liquid chromatography, to ensure higher purity of the samples, or by implementing tandem mass spectrometry, resulting in higher specificity [82]. Alone, MS does not suffice as a method for neo-antigen identification as it requires a priori knowledge of the potential target, although exceptions can be made for peptides spliced in the proteasome, peptides with post-translational modification (PTM) and peptides from non-coding regions [83]. However, because of its high sensitivity, accuracy and reproducible qualification and quantification of the HLA peptidome, it makes for a valid approach for neo-antigen validation.

In contradiction to these arguments, there have been few reports of neo-antigen identification using MS as a stand-alone method. These include the identification of the tumor ligandome using MS followed by matching with exome and transcriptome data, mostly in murine tumor models [84] or human tumor cell lines [85,86,87], although this approach for neo-antigen identification in human mantle cell lymphoma [88] and human melanoma [74,86] has been reported as well.

Although MS is important for validation of candidate neo-antigen libraries that are generated based on NGS and bioinformatics, it still faces challenges, mostly related to sample preparation. One challenge is the isolation of HLA bound peptides that are present in low abundance. High HLA expression and high cell numbers, ranging from 1*10^8^ to 5*10^8^ cells per sample, are required for MS-based analysis of the immunopeptidome [38,86]. Therefore, efficient isolation of HLA-peptide complexes is key. Common methods for immunopeptidome enrichment are immunoprecipitation (IP) of HLA-peptide complexes and/or mild acid elution (MAE) of HLA bound peptides [89]. IP is regarded as highly specific and flexible, allowing HLA-peptide isolation from a range of biological samples upon MAE. Although MAE without IP is a more time- and cost-effective approach, it lacks selectivity, as up to 60% of the eluted peptides can be contaminants [83]. Lanoix et al. [90] compared both methods and concluded that both resulted in reproducible candidate neo-antigen libraries. However, each method gave rise to a different spectrum of peptides, exemplified by the fact that the neo-antigen library generated using MAE resulted in more neo-antigens caused by PTM. Their analysis was concluded with the suggestion that both approaches should be combined for immunopeptidomics.

A shortcoming of the in silico genomic and peptidomic workflows is their inability to guarantee immunogenicity of the presented neo-epitopes [77,80,81]. Different strategies can be used to test immunogenicity. For example, Chen et al. [91] stimulated patients’ peripheral blood mononuclear cells (PBMCs) with neo-epitopes, evaluating T cell activation by IFN-γ ELISPOT and flow cytometry, assessing 4-1BB (CD137) upregulation. Additionally, Perumal et al. [92] used patients’ PBMCs, which were non-specifically stimulated for 14–21 days prior to specific stimulation with de candidate neo-epitope. T cell reactivity was assessed with intracellular cytokine staining, tetramer staining, TCR sequencing and cytotoxic killing assays. However, these T cell reactivity screenings are laborious. High throughput screening of candidate neo-epitopes, which would improve implementation of neo-antigen vaccines into clinical practice, are, therefore, necessary. Such screening could be attempted using reporter cells that are modified to express chimeric receptors [93,94,95,96] A first example is the use of signaling and antigen-presenting bifunctional receptors (SABRs) that consist of an HLA molecule tethered to a peptide and fused to an intracellular CD3ζ and a CD28 co-stimulatory signaling domain. These SABRs are transferred to Jurkat cells that are modified to express green fluorescent protein (GFP) in response to CD3ζ signaling. Thus, when the SABR is bound by a cognate TCR, the CD3ζ domain of the SABR is activated, triggering GFP as well as CD69 expression. The GFP and CD69 double positive Jurkat cells can be sorted for further identification and sequencing of the recognized peptide [93]. In a similar approach, Kisielow et al. [94] used an MHC-TCR chimeric construct, referred to as MCR, and designed for the identification of tumor-specific peptides recognized by CD4^+^ T cells. The MCR libraries were generated by cloning fragmented tumor cell cDNA into MCR sequences and were used in a similar way as SABRs. Easy implementation of these high throughput screening methods has to be proven, and the advantages of these methods should outweigh potential disadvantages. Nonetheless, it is conceivable that these new methods become best practice for unbiased identification of T cell targeted neo-antigens in cancer patients.

## 4. History of mRNA-Based Cancer Vaccines 

**Figure 3:**
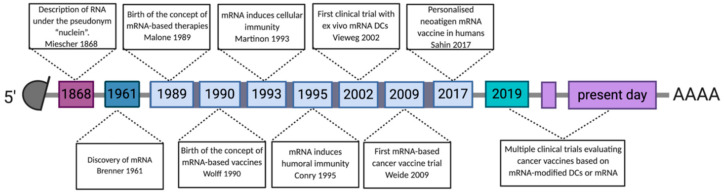
Timeline showing discoveries and advances in the development of mRNA-based cancer vaccines. Abbreviations: 3′ poly-A, three prime polyadenylic acid tail; 5′ cap, five prime cap; mRNA, messenger RNA; LNP, lipid nanoparticles; DCs, dendritic cells.

The discovery of RNA began in 1868 with the description of nucleic acids by Friedrich Miescher, who called this the ‘nuclein’ after its location in the nucleus (Figure 3) [97]. In 1961, Brenner et al. [98] described mRNA as an unstable intermediate carrying encoding information from genes to ribosomes for protein synthesis. Twenty-eight years later, mRNA was upgraded to a high potential vehicle for in vitro delivery of proteins to human, rat, mouse, Xenopus and Drosophila cells, as proven by the delivery of firefly luciferase [99]. Wolff et al. [100] described in 1990 that injection of naked mRNA in mouse skeletal muscles resulted in transfection of muscle cells with protein expression lasting for several days. This study delivered a proof-of-concept for the use of IVT mRNA to deliver genetic information to produce proteins in cells in situ. Despite encouraging results, many concerns associated with IVT mRNA instability, immunogenicity and low in vivo transfection efficiency, discouraged the research community to further work on this technology platform for vaccine development. In the mid-1990s, two pioneers, Martinon and Conry, validated IVT mRNA for vaccination purposes, showing induction of influenza specific CTL-responses [101] or carcinoembryonic antigen specific antibodies [102] after in vivo delivery of IVT mRNA. From this point onward, IVT mRNA received more and more attention as a vaccine technology platform, not only in the context of cancer, but also in the context of infectious diseases, as evidenced by the rise of companies investing in IVT mRNA vaccines in the last two decades (Table 1).

Two approaches of using IVT mRNA in cancer vaccines can be pursued. The first approach is to transfer IVT mRNA encoding TAAs into the patient’s DCs ex vivo [1]. These transfected cells are then administered back to the patient. A first clinical trial using ex vivo DCs transfected with IVT mRNA encoding prostate specific antigen (PSA) was performed at the turn of the century. The mRNA-based DC vaccine was administered via intravenous injection to patients with prostate cancer using a dose escalation regimen [103]. This initial clinical trial was promising, as it showed feasibility, lack of toxicity, immunogenicity and signs of clinical efficacy. More specifically, dose-limiting toxicity or adverse effects, such as autoimmunity, were not observed, while PSA-specific T cells were detected in all patients. Clinically, six out of seven patients experienced a significant decrease in the log slope PSA with three evaluable patients exhibiting a transient molecular clearance of circulating tumor cells. In the following years, over 20 clinical trials were published with autologous DCs that were transfected with IVT mRNA encoding TAAs in various indications among which melanoma [8,104,105,106,107,108,109], uterine cancer [110], ovarian cancer [111,112], pancreatic cancer [113,114], colorectal cancer (CRC) [115], prostate cancer [107], renal cell carcinoma [116], hepatocellular carcinoma [117], glioblastoma [118,119,120], acute myeloid leukemia [121,122,123] and multiple myeloma [124]. These studies proved the feasibility and safety of the mRNA-based DC vaccination approach; however, overall clinical benefit exceeding 25% was rarely reported. In this context, it is interesting to mention that one of the vaccines reaching this threshold was the TriMixDC-vaccine, consisting of monocyte-derived DCs electroporated with TAA mRNA in combination with three mRNA molecules encoding CD40 Ligand (CD40L), constitutively active Toll-like receptor (TLR) 4 (caTLR4) and CD70, highlighting that mRNA is an interesting cancer vaccine technology platform allowing the transfer of tumor antigens to DCs in combination with proteins that endow the DCs with strong T cell stimulatory capacity [125]. An in-depth review on therapeutic cancer vaccination with ex vivo RNA transfected DCs was recently published by Dörrie et al. [23].

In the second approach, IVT mRNA is injected directly into the patient, circumventing ex vivo production of patient-specific DCs. In this context, the study by Weide et al. [126], dating back to 2009, is a hallmark study showing that intradermal injection of IVT mRNA, stabilized with protamine sulphate and encoding several TAAs (Melan-A, Tyrosinase, gp100, MAGE-A1/A3, survivin) is feasible and safe and activates T cells. In particular, 21 patients with metastatic melanoma received the vaccine together with granulocyte macrophage colony-stimulating factor, a growth factor that stimulates myelopoiesis. Adverse events that were observed, however, were not higher than grade 2. Four out of seven patients were immunologically evaluable. Of these, half showed an increase in vaccine-specific T cells. Moreover, one patient with measurable disease showed a complete response. Since that time, issues with instability, inefficient in vivo delivery, inefficient translation of IVT mRNA as well as immunogenicity of IVT mRNA have been addressed and mostly overcome, as reviewed in depth elsewhere [37,127]. We will describe some of these issues and their solution in the context of mRNA as a technology platform for neo-antigen vaccination, showing that mRNA-based vaccines have reached the status of high potency vaccines with the potential of fast development, low-cost manufacturing and safe administration to patients.

## 5. mRNA as a Platform for Neo-Antigen Vaccination

In vitro-transcribed mRNA represents an innovative modality to deliver neo-antigens to APCs in principle both in vitro and in vivo. This approach offers many advantages with regard to speed of development, production, scalability, reliability, manufacturing costs and efficacy, thereby positioning mRNA as a prime candidate for preparing cancer vaccines [128].

Neo-antigen mRNA should be delivered to the cytoplasm of APCs, preferably DCs, where it can be translated into protein for further processing and antigen presentation to T cells. Various strategies have been developed for the delivery of IVT mRNA to DCs that have been generated in the laboratory (ex vivo) or that are present within the cancer bearing subject (in vivo). These delivery methods range from the addition of mRNA simply dissolved in a buffer of choice (e.g., lactated Ringer’s solution) [129,130,131,132], to more sophisticated strategies that facilitate mRNA uptake through its encapsulation in carriers, such as lipid and/or polymer-based nanoparticles [133,134,135], cell penetrating peptides [136] or the addition of mRNA in solution, which can leak into the cells upon their electroporation [137,138] or sonoporation [139]. These different strategies have been reviewed elsewhere [1,24,134]. Inside the cytoplasm, the IVT mRNA is translated into the encoded protein, which might be post-translationally modified. However, mRNA translation can be curtailed, as a result of the IVT mRNA delivery itself [140]. This is a result of recognition of the IVT mRNA by a number of innate RNA sensors, in particular when the IVT mRNA is produced with natural nucleotides and when capping is incomplete. These innate RNA sensors include endosomal TLRs (TLR3, 7, 8) and cytosolic RNA receptors (retinoic acid-inducible gene I, melanoma differentiation-associated protein 5) [141,142,143,144,145]. Innate RNA sensing drives the expression of type I IFNs, such as IFN-α/-β, and pro-inflammatory cytokines, which in essence act as a double-edged sword. These cytokines create an environment that is ideal for T cell activation; however, they also downregulate the translation of mRNA, and expedite its degradation [37,146]. This has a major impact on the availability of the encoded antigen and negatively affects T cell activation [131,147]. Seminal work from Kariko et al. [148] showed that endogenous mRNA showed less immune activating capacity than IVT mRNA, which was attributed to natural post-translational modifications of nucleotides. This observation led to the widespread use of methylated nucleosides and/or pseudouridine for the production of mRNA that shows reduced immune activating capacity with enhanced stability and translational capacity [25].

mRNA molecules encoding different antigens can be included in one vaccine [133,134], and multimeric antigens that are more difficult to manufacture with traditional technologies can be produced [50]. mRNA is translated into the encoded protein in the DCs. The resulting protein is further processed into small peptides for presentation on HLA-I proteins to CD8^+^ T cells, holding the promise of stimulating broad CTL responses. Co-stimulation needs to be provided together with antigen presentation. Next-generation mRNA vaccines are generated with chemically modified nucleotides; therefore, they lack intrinsic immune activating capacity, signifying the need for adjuvants. Examples of adjuvants that have shown merit in mRNA-based cancer vaccines are monophosphoryl lipid A [135], α-Galoctosylceramide [3], RNActive [149] and mRNA encoding DC potentiating proteins, exemplified by but not limited to TriMix mRNA [125,150]. These adjuvants operate in different ways; however, they ensure that the mRNA-modified DCs adopt a mature phenotype and function. Second, the activation of CTLs needs to be supported by CD4^+^ T_H_1 cells to ensure full functionality of these CTLs [54]. Presentation of antigens to CD4^+^ T cells occurs in MHC-II molecules that are loaded in MIIC compartments with peptides that are derived from exogenous antigens. When using mRNA as an approach to deliver antigens, it is imperative to guide the antigen to these compartments through the coupling of the antigenic sequence to MHC-II targeting signals, such as the signal sequence of the invariant chain, lysosomal-associated membrane protein (LAMP) or DC-LAMP [2,54,55]. Activated CD4^+^ T_H_1 cells affect DCs in several ways ensuring CTL activation, among others through CD40-CD40L mediated DC licensing [151] and production of IFN-γ [152], both stimulating IL-12 production. Notably, CD4^+^ T cells can exert cytotoxicity and as such can direct cancer cell rejection in the absence of CTLs [4,5]. With regard to neo-antigen vaccines, a vast majority of immunogenic neo-epitopes is recognized by CD4^+^ T cells in tumor-bearing C57BL/6 mice [50]. This suggests a key role for CD4^+^ T cells in the neo-antigen directed anti-tumor immune response. This is further supported by the results of recently published neo-antigen vaccine trials that show the presence of neo-antigen specific CD4^+^ T cells, even though vaccination was performed with neo-epitopes that were predicted to bind to MHC-I [19,20,21].

mRNA vaccine production starts with the design of the antigen in silico, offering the advantage of rapid production and preclinical evaluation, which in view of neo-antigens could entail evaluation of the correct presentation of neo-epitopes through peptidomics and/or evaluation of the neo-epitope’s immunogenicity. This can accelerate selection of suited neo-antigens for personalized cancer vaccination. Despite a different sequence for each neo-antigen, the production process is standard, reducing the timing and cost for vaccine production due to common manufacturing processes and infrastructure. The production process begins with cloning and linearization of the DNA template to produce multiple copies of the coded mRNA using RNA polymerases (T3, T7, SP6).

Chemically modified nucleotides are often used as building blocks to lower sensing by PRRs and to enhance translational efficacy [32,153]. Adding a 5′ cap and the 3′ poly-A tail can be done during or after (enzymatically) the in vitro transcription reaction [154,155,156,157]. The DNA template is removed using the enzyme DNase and the IVT mRNA is purified using LiCl/NaCl-EtOH precipitation or using dT microbeads [158]. Cellulose [159] or HPLC [32] purification, or RNase III enzymatic treatment [160], can be performed to remove contaminants, such as double stranded RNA (dsRNA), that result from RNA polymerase overactivity [161,162]. This also lowers PRR sensing and enhances translational efficacy. Notably, IVT mRNA can be produced according to good manufacturing practice (GMP), as plasmid DNA, enzymes and other reagents are available from commercial providers as GMP-grade starting materials. The IVT mRNA production process can be standardized, allowing rapid production of any protein of interest, making this approach ideally suited for incorporation into the neo-antigen vaccine pipeline.

## 6. Studies with Neo-Antigen mRNA

To our knowledge, clinical trials with DCs that have been loaded ex vivo with neo-antigen mRNA have not yet been performed. However, the Flemish-based consortium “Persomed” has set up a roadmap to develop such a vaccine for the treatment of patients with CRC (Table 1), leveraging the knowledge gathered by Thielemans et al. [8,105,106,163,164,165] in producing potent, clinical-grade, IVT mRNA-electroporated DC vaccines. Meanwhile, several players in the IVT mRNA field are studying the direct delivery of neo-antigen mRNA to APCs in situ [23,24].

The results of a pioneering study by Sahin et al. [19] have been reported. They determined the mutanome, a term used to describe the detection and mapping of somatic mutations of the tumor’s genome, of 13 stage III/IV melanoma patients. Non-synonymous mutations were identified by comparative whole genome/exome and RNA sequencing of tumor biopsies and healthy blood cells. Bioinformatics was used to rank the mutations according to the predicted affinity for binding to the patient’s HLA-I as well as HLA-II molecules. Except for one patient, an mRNA vaccine consisting of two IVT mRNA molecules, each encoding five linker-connected 27-mer peptides with the mutation in position 14, referred to as a pentatope, was produced. All patients were vaccinated at an mRNA dose of 0.5 or 1 µg per vaccination round with a maximum of 20 vaccine doses by percutaneous injection of the mRNA neo-antigen vaccine into the inguinal lymph node. During the period of vaccine production, patients with tumors expressing the TAAs, NY-ESO-1 or tyrosinase, received a previously described mRNA-based vaccine encoding these TAAs [166]. The results of this neo-antigen mRNA vaccine study are encouraging. Eight patients, who had no radiologically detectable tumors at the start of neo-epitope vaccination, remained recurrence-free in the 12- to 23-month follow-up period. Five patients had metastatic disease at the start of vaccination. Two of them experienced objective responses, and a third patient developed a complete response as a result of combined therapy with a programmed death-1 (PD-1) blocking antibody. Immune monitoring showed that all patients developed T cell responses against neo-epitopes included in the vaccine with responses observed against 60% of the included neo-epitopes. It was, however, reported that one patient with metastatic disease, who benefited at first, developed resistance to T cell mediated tumor cell killing. As a follow up to this pioneering trial, this team is performing a next clinical trial with a mutanome-based mRNA vaccine in triple negative breast cancer (TNBC) [167]. In this trial, the mRNA is formulated in a lipid nanoparticle (LNP) and will be delivered intravenously. A query on www.clinicaltrials.gov showed that 12 clinical trials on direct delivery of neo-antigen mRNA to APCs in situ are currently ongoing, including the study in TNBC (Table 2). In these studies, IVT mRNA is administered using various routes of delivery either formulated in LNP or merely dissolved in an appropriate buffer (naked mRNA). This begs the question: “How will these different administration routes and formulation strategies affect the vaccine efficacy?”

## 7. Future Perspectives

### 7.1. Neo-Antigen Prediction and Prioritization

Implementation of NGS and bioinformatics in the neo-antigen identification workflow has led to discovery of the source of neo-antigens, including cancer specific overexpression, alternative exon splicing, intron retention, gene fusions in addition to SNVs and INDELs [168]. MS and T cell reactivity studies have confirmed these findings. However, it is important to mention that the fraction of MS validated neo-antigens, compared to the libraries predicted with bioinformatics, is rather limited. This is likely due to the systematic bias of in silico predictions, combined with biological unknowns in antigen processing. Neo-antigen prediction tools usually have a bias as a result of the training algorithm used. For example, NetMHCpan was trained using viral epitopes from the Immune Epitope Database, resulting in a selection bias towards viral-like neo-antigens. There is some evidence that neo-epitopes with homology to microbial epitopes are immunogenic [15,61]. Nonetheless, this selection bias withholds the risk of neglecting other potentially interesting neo-antigens. Furthermore, multiple training sets are available for frequent HLA types (e.g., HLA-A2), while training sets for infrequent HLA types are largely missing [74,168]. Implementing biological aspects of protein-to-peptide processing and HLA-peptide binding into the training algorithms is another strategy to improve in silico prediction of valid neo-epitopes. Some tools are available that consider peptide cleaving and processing of proteins by the immunoproteasome, including NetChop20S, NetChopCterm, and ProteaSMM for HLA-I presentation and PepCleaveCD4 and MHC NP II for HLA-II presentation. Different methods that take aspects of peptide loading into account, for example affinity of the antigen-derived peptide for the transporter of antigen processing, are also in development [169]. In general, an optimal prediction tool is likely to arise from continued improvement and revision of existing prediction algorithms. This can be achieved through continuous comparison of data obtained via bioinformatics, MS and neo-epitope immunogenicity testing. The increase in data resulting from neo-antigen vaccination trials will certainly facilitate improving and refining the prediction algorithms used for neo-antigen identification [170]. Moreover, shared neo-antigens, resulting from oncogenic driver mutations, are being discovered among cancer patients in several cancer types [171]. These shared neo-antigens could be of interest to manufacture a more broadly applicable neo-antigen cancer vaccine. An example hereof is the mRNA vaccine encoding the four most common KRAS mutations, rereferred to as mRNA-5671. This vaccine is in phase I clinical testing for patients with KRAS-mutant colon, pancreatic or lung cancer, as a monotherapy or in combination with pembrolizumab, PD-1/PD-L1 blocking therapy (NCT03948763). In time more of these shared neo-antigens will be identified across both patients and tumor types, facilitating to more rapidly move forward with neo-antigen mRNA vaccines [172,173].

### 7.2. Neo-Antigen Immunogenicity Screening

Assays that evaluate T cell reactivity have been developed to determine the immunogenicity of candidate neo-antigens. Up-regulation of T cell activation markers such as 4-1BB or production of IFN-γ have been determined as a measure of the T cells specificity for presented neo-epitopes [91]. Additionally, staining of TCRs with tetramers followed by selection of these T cells and TCR sequencing has been performed [92]. However, these methods are not highly sensitive and depending on the stimulation protocol, high background can occur and can make interpretation difficult. Therefore, Danilova et al. [174] introduced a more sensitive method for screening of neo-epitope reactive T cells. In this method sequencing of the TCR of short-term, peptide-stimulated T cell cultures is combined with a bioinformatics platform to identify antigen specific clonotypic amplification. Chimeric receptors, SABRs and MCRs, which contain a candidate neo-epitope tethered to an antigen-presenting molecule fused to a CD3ζ domain and are expressed in NFAT-GFP reporter cells, have been used as an alternative to the screening of T cells as well [93,94]. Binding of the neo-epitope in the antigen-presenting molecule by a cognate TCR results in GFP expression in the Jurkat reporter cells, allowing their selection for subsequent neo-epitope sequencing. Such a reporter-based assay could enable unbiased and high throughput identification of T cell targeted neo-epitopes. This could be achieved using mRNA as a platform for chimeric antigen expression in these reporter cells, since mRNA encoding these chimeric receptors is prepared faster than viral vectors, the method currently used for reporter cell modification, and since mRNA can be used to enable protein expression in these reporter cells [175,176].

### 7.3. Monitoring the Efficacy of Neo-Antigen Vaccines?

Clinical trials with neo-antigen mRNA vaccines are mostly in early stages (Table 2). For these, safety and tolerability of the vaccine (often escalating doses) is the primary objective. However, evaluation of immune activity (so-called immune monitoring) and tumor response (e.g., biomarkers detection) could be addressed early on and continued in pivotal trials. This would provide additional information on the success or failure of the neo-antigen vaccine in individual patients. This is important for several reasons. The magnitude of the response to the vaccinal neo-antigens will vary from patient to patient. This complicates comparing clinical outcomes between patients. However, during neo-antigen discovery, neo-antigens are scored on predicted immunogenicity using parameters such as HLA affinity, hydrophobicity, etc. Measuring immune activation could allow correlation of the magnitude of the response to the neo-antigen’s immunogenicity score and might reveal a minimal immunogenicity score to which a successful neo-antigen should adhere. Moreover, numerous mechanisms of tumor cell resistance to a T cell mediated attack are described. These could hamper clinical benefit even though neo-antigen specific T cells were elicited. Currently, there are data that support the idea of combining neo-antigen vaccination with other (immuno-) therapies.

### 7.4. Combining with Other Immunotherapies

Tumors develop mechanisms of resistance when under pressure of a T cell mediated attack. The pioneering study of Sahin et al. [19] confirmed that upon an attack by neo-antigen specific T cells, tumors develop resistance mechanisms, as exemplified by the outgrowth of β2-microglobulin-deficient melanoma cells in one patient, and the need for additional PD-1 blockade to achieve complete response in another patient.

It is generally accepted that combining different immunotherapies is likely allowing the immune system to outsmart the tumor. In this regard, neo-antigen mRNA-vaccines that induce T cells against multiple neo-epitopes are likely to benefit from strategies that support the vaccine-induced T cells or that activate other immune effectors with complementary activity. T cells that infiltrate the TME can be confronted with a plethora of immunosuppressive cells and molecules. Therefore, strategies that reverse immune suppression are prime candidates for a combination with neo-antigen mRNA-vaccines. As described above, complete remission has been achieved in a patient with advanced, metastatic melanoma by combining neo-antigen mRNA vaccination with PD-1 inhibition [19]. Activation of immune effectors with complementary activity could be achieved in various ways. For instance, in the case of CD1d-positive tumors, one could include α-Galactosylceramide in the mRNA-vaccine formulation to stimulate NKT cells [3]. Another strategy that could be envisaged is targeting of antigens on the surface of tumor cells to activate complement, macrophages or NK cells, in this way attacking tumor cells from multiple angles, making escape from immunity more difficult [177]. In addition, the use of chemotherapy and/or radiotherapy could be of interest as these strategies have the ability to alter the composition of the tumor immune microenvironment. In study NCT4161755, the neo-antigen mRNA vaccine and atezolizumab are combined with the chemotherapeutic drug mFOLFIRINOX. This drug has been shown to increase CTLs, while lowering regulatory T cells; therefore, they could support the neo-antigen-specific CTLs activated by the neo-antigen mRNA vaccine [178]. An extended review on strategies that are explored in combination with neo-antigen vaccines in general, including mRNA based neo-antigen vaccines, is provided elsewhere [179].

## 8. Conclusions

Cancer vaccines based on neo-antigens hold the promise of improved tumor specificity and immunogenicity compared to cancer vaccines based on TAAs. The IVT mRNA platform could be exploited for immunogenicity testing of candidate neo-epitopes, for example by its incorporation in reporter assays using chimeric receptors, as well as for neo-antigen vaccination itself, being able to deliver neo-epitopes in addition to proteins that support immune activation. Currently, clinical experience with mRNA-based neo-antigen vaccines is scarce. However, data from the first clinical trials are encouraging and warrant further exploration of mRNA as a neo-antigen vaccine platform. The results from different ongoing clinical studies will add to our understanding on the pre-requisites for a potent neo-antigen mRNA-vaccine, potentially shedding light on a preferred route of administration as well as a preferred method of mRNA formulation, lyophilized or dissolved in an appropriate buffer, encapsulated in nanoparticles or already transferred to DCs ex vivo. Overall, results from ongoing clinical trials will catalyze a movement for therapeutic neo-antigen mRNA-vaccines.

## Figures and Tables

**Table 1:** Companies that exploit IVT mRNA for cancer vaccine purposes. Abbreviations: mRNA, messenger RNA; DC, dendritic cell; moDC, monocyte-derived DC.

Company or Consortium	Start Date	Country	Core Business	Website
TriLink	1996	USA	Contract development and manufacturing of mRNA medicines	https://www.trilinkbiotech.com/
Curevac	2000	Germany	mRNA medicines, including cancer vaccines	https://www.curevac.com/
BioNtech	2008	Germany	mRNA medicines, including neo-antigen cancer vaccines	https://biontech.de/
ModeRNA	2010	USA	mRNA medicines for a wide range of diseases and conditions, including cancer	https://www.modernatx.com/
eTheRNA	2013	Belgium	mRNA medicines to treat infectious diseases and cancer, including neo-antigen vaccines	https://www.etherna.be/
Kernal Biologics	2016	USA	mRNA medicines to treat infectious diseases and cancer	https://www.kernalbio.com/
Stemirna Therapeutics	2016	China	mRNA medicines to treat infectious diseases and cancer, including neo-antigen vaccines	http://www.stemirna.com/
Persomed	2020	Belgium	DC vaccines based on moDCs modified with neo-antigen and DC activating mRNA	https://www.persomed.be/

**Table 2:** Clinical trials in which neo-antigen mRNA is an investigational medicinal product. Abbreviations: i.d., intradermal; i.m., intramuscular; i.n., intranodal; i.v., intravenous; LNP, lipid nanoparticle; NA, not applicable; NN, not known, NSCL, non-small cell lung cancer; s.c., subcutaneous; TAA, tumor-associated antigen; TNBC, triple negative breast cancer.

Cancer Type	Study	Phase	Formulation	Route	Other Therapy	Response
TNBC	NCT02316457	Phase I	LNP	i.v.	NA	Study extended
Melanoma	NCT02035956	Phase I	Naked mRNA	i.n.	RBL001/RBL002	Not published
NSCLC	NCT03164772	Phase I/II	LNP	i.d.	Durvalumab, Tremelumumab	Study ongoing
Solid tumors	NCT03289962	Phase I	Naked mRNA	i.v.	Atezolizumab	Study ongoing
Solid tumors	NCT03313778	Phase I	LNP	i.d.	Pembrolizumab	Study ongoing
Solid tumors	NCT03480152	Phase I/II	Naked mRNA	i.m.	NA	MTD not reached
Solid tumors, lymphoma	NCT03468244	NN	Naked mRNA	s.c.	NA	Study ongoing
Metastatic melanoma	NCT03815058	Phase II	LNP	i.v.	Pembrolizumab	Study ongoing
High-risk of recurrence melanoma	NCT03897881	Phase II	NN	NN	Pembrolizumab	Study ongoing
Esophageal cancer, NSCLC	NCT03908671	NN	LNP	s.c.	NA	Study ongoing
Pancreas cancer	NCT04161755	Phase I	NN	NN	Atezolizumab, chemotherapy	Study Ongoing
NSCLC	NCT04267237	Phase I	LNP	i.v.	Atezolizumab	Study ongoing
KRAS-mutant pancreas, colon and lung cancer	NCT03948673	Phase I	NN	i.m.	Pembrolizumab	Study ongoing

## Abbreviations

3′ poly-A	three prime polyadenylic acid
5′ cap	five prime cap
APC	antigen-presenting cell
CD40L	CD40 ligand
CRC	colorectal cancer
CTL	cytotoxic T lymphocyte
DC	dendritic cell
GFP	green fluorescent protein
GMP	good manufacturing practice
HLA	human leukocyte antigen
i.d.	intradermal
i.m.	intramuscular
i.n.	intranodal
i.v.	intravenous
IFN	interferon
INDEL	insertion and deletion
iNKT	invariant natural killer T cell
IP	immunoprecipitation
IVT	in vitro transcribed
LAMP	lysosomal-associated membrane protein
LNP	lipid nanoparticle
MAE	mild acid elution
MCR	MHC-TCR chimeric receptor
MHC	major histocompatibility complex
moDC	monocyte-derived dendritic cell
mRNA	messenger RNA
MS	mass spectrometry
NGS	next-generation sequencing
NSCL	non-small cell lung cancer
PBMC	peripheral blood mononuclear cell
PD-1	programmed death-1
PRR	pattern recognition receptor
PSA	prostate specific antigen
PTM	post-translational modification
RNases	ribonucleases
s.c.	subcutaneous
SABR	signaling and antigen-presenting bifunctional receptor
SNV	single nucleotide variants
TAA	tumor-associated antigen
TCR	T cell receptor
T_H_1	T helper 1 cell
TLR	toll-like receptor
TNBC	triple negative breast cancer
UTR	untranslated region
